# Prediction of Transcriptional Terminators in *Bacillus subtilis* and Related Species

**DOI:** 10.1371/journal.pcbi.0010025

**Published:** 2005-08-12

**Authors:** Michiel J. L. de Hoon, Yuko Makita, Kenta Nakai, Satoru Miyano

**Affiliations:** Human Genome Center, Institute of Medical Science, University of Tokyo, Minato-ku, Tokyo, Japan; Weill Medical College of Cornell University, United States of America

## Abstract

In prokaryotes, genes belonging to the same operon are transcribed in a single mRNA molecule. Transcription starts as the RNA polymerase binds to the promoter and continues until it reaches a transcriptional terminator. Some terminators rely on the presence of the Rho protein, whereas others function independently of Rho. Such Rho-independent terminators consist of an inverted repeat followed by a stretch of thymine residues, allowing us to predict their presence directly from the DNA sequence. Unlike in *Escherichia coli,* the Rho protein is dispensable in *Bacillus subtilis,* suggesting a limited role for Rho-dependent termination in this organism and possibly in other *Firmicutes*. We analyzed 463 experimentally known terminating sequences in *B. subtilis* and found a decision rule to distinguish Rho-independent transcriptional terminators from non-terminating sequences. The decision rule allowed us to find the boundaries of operons in *B. subtilis* with a sensitivity and specificity of about 94%. Using the same decision rule, we found an average sensitivity of 94% for 57 bacteria belonging to the *Firmicutes* phylum, and a considerably lower sensitivity for other bacteria. Our analysis shows that Rho-independent termination is dominant for *Firmicutes* in general, and that the properties of the transcriptional terminators are conserved. Terminator prediction can be used to reliably predict the operon structure in these organisms, even in the absence of experimentally known operons. Genome-wide predictions of Rho-independent terminators for the 57 *Firmicutes* are available in the Supporting Information section.

## Synopsis

In prokaryotes, genes belonging to the same operon are transcribed in a single mRNA molecule. Transcription starts as the RNA polymerase binds to the promoter and continues until it reaches a transcriptional terminator. To understand the gene regulatory network of transcription in bacteria, it is important as a first step to determine the operon structure. In this paper, the authors show that (unlike in *Escherichia coli*) most terminators in *Bacillus subtilis* function independently of the terminator protein Rho. As these Rho-independent terminators consist of an inverted repeat followed by a stretch of thymine residues, their presence can be predicted directly from the DNA sequence. The authors derived a decision rule by analyzing experimentally known terminating sequences in *B. subtilis,* and show that the operon boundaries can be found with a high accuracy (about 94%) in *B. subtilis* and other *Firmicutes,* even in the absence of experimentally known operons in the given organism. The properties of the transcriptional terminators are shown to be conserved within the *Firmicutes* phylum. For bacteria other than *Firmicutes,* the prediction accuracy is considerably lower, suggesting that Rho-dependent or possibly currently unknown termination mechanisms are important in these organisms.

## Introduction

Since the sequencing of the first bacterial genome, the proteobacterium *Haemophilus influenzae* [1], the complete genomes of 213 microbial organisms have been sequenced, while the sequencing of many more microbial genomes is under way. The availability of these microbial genomes allows us to predict the function of genes in less-characterized bacteria based on their homology to well-studied organisms, such as *Escherichia coli* and *Bacillus subtilis*. Similarly, one may attempt to predict the transcriptional regulation of genes in less-characterized organisms using existing knowledge of gene regulation in *E. coli* and *B. subtilis*.

Operons, a group of adjacent genes on the same strand of DNA that are transcribed into a single mRNA molecule, form the basic unit of transcription in prokaryotes. Transcription starts from a promoter upstream of the first gene and continues until the RNA polymerase reaches a terminator structure downstream of the last gene in the operon.

Since genes on the same strand belonging to different operons are separated by a terminator followed by a promoter, the operon structure of a bacterial genome can be predicted by the space in base pairs between the genes [2,3]. However, an analysis of the DBTBS database of transcriptional regulation in *B. subtilis* [4] revealed that more than 20% of its genes in known polycistronic operons are transcribed from more than one promoter. These additional promoters are often located downstream of the first gene, such that only part of the operon is transcribed from the internal promoter. Similarly, we found that almost 6% of the known polycistronic operons contain an internal read-through terminator, at which partial continuation of transcription occurs. The existence of such internal promoters and terminators complicates the definition of an operon.

Transcriptional units can be defined more precisely by the location of the promoters and the transcriptional terminators. Previously, a prediction of transcriptional units of *E. coli* by searching for promoters and terminators using hidden Markov models yielded an accuracy of about 60% [5]. In this paper, we consider the prediction of transcriptional terminators in *B. subtilis* and related species, in particular those of the *Firmicutes* phylum, to which *B. subtilis* belongs. This phylum consists of a heterogeneous group of mostly Gram-positive bacteria whose genomes have a low G+C content. Several important disease-causing organisms belong to the *Firmicutes* phylum, such as *Clostridia, Streptococci, Staphylococci,* and *Mycoplasmas,* as well as important industrial microbes such as the *Lactobacilli*.

In the Gram-negative *E. coli,* belonging to the phylum of *Proteobacteria,* transcriptional termination is achieved by Rho-independent terminators, which can function in vitro, and Rho-dependent terminators, which need the protein Rho to be present to be functional [6]. Most bacteria contain a protein homologous to *E. coli*'s Rho; notable exceptions are the *Firmicutes* *Mycoplasma genitalium* and *M. pneumoniae,* *Streptococcus pneumoniae* and *S. pyogenes,* and *Ureaplasma urealyticum,* and the cyanobacterium *Synechocystis sp.* strain PCC6803 [7–9]. However, the relative importance of Rho-dependent termination varies between bacteria. In the proteobacteria *E. coli* and *Rhodobacter sphaeroides,* as well as the actinobacterium *Micrococcus luteus,* Rho is essential [10–12]; in the proteobacterium *Caulobacter crescentus,* Rho is required for oxidative stress survival [13]. Also, an analysis of the average RNA folding energy near stop codons suggested that no stem-loops are formed in the *Firmicutes* *Mycoplasma genitalium* and *M. pneumoniae,* the actinobacterium *Mycobacterium pneumoniae,* the cyanobacterium *Synechocystis sp.* PCC 6803, the proteobacterium *Helicobacter pylori,* the spirochetes *Treponema pallidum* and *Borrelia burgdorferi,* the aquifica *Aquifex aeolicus,* and the euryarchaeota *Methanococcus jannaschii,* *Methanobacterium thermoautotrophicum,* *Archaeoglobus fulgidus,* and *Pyrococcus horikoshii,* implying that Rho-independent termination does not play a significant role in these organisms [14]. On the other hand, in the Gram-positive *B. subtilis* the Rho protein is dispensable [15], suggesting that Rho-independent termination is dominant in this organism. Indeed, the only known case of Rho-dependent termination in *B. subtilis* is the *rho* gene itself. Similarly, Rho is not essential for viability or virulence in the Gram-positive bacterium *Staphylococcus aureus* [9]. Furthermore, Gram-positive bacteria (except for *Micrococcus luteus* [12]) being resistant to the Rho-inhibiting antibiotic bicyclomycin previously led to the suggestion that Rho is usually dispensable in these bacteria [9].

Rho-independent terminators consist of an inverted repeat in the primary DNA sequence, followed by a short stretch of thymine residues. The inverted repeat gives rise to a stem-loop structure in the transcribed mRNA molecule, which halts the RNA polymerase complex. The decreased binding of the uridine stretch of the nascent RNA polymerase complex to the corresponding adenine stretch in the DNA causes the polymerase to dissociate from the DNA, terminating transcription. Experimentally, the presence of a transcriptional terminator is often established by measuring the mRNA length in a Northern blotting experiment, which is usually not precise enough to determine the exact termination site. In experiments in which the termination site was determined in a primer extension experiment of the 3′ end of the mRNA, termination was shown to occur at or near the T-stretch following the stem-loop. Rho-independent terminators in *E. coli* can be distinguished reliably from intracistronic sequences and random sequences from the Gibbs free energy of stem-loop formation and the properties of the T-stretch [16,17]. The properties of Rho-dependent terminators are less well-studied.

The apparent dominance of Rho-independent termination in *B. subtilis* and *Staphylococcus aureus* and the feasibility of distinguishing Rho-independent terminators from random and intracistronic sequences suggests that prediction of transcriptional terminators may be a reliable method to predict operons in these and related organisms. However, our current knowledge of transcriptional terminators does not suffice for such a prediction. First, it is unclear if the properties of transcriptional terminators as found in *E. coli* are conserved in other prokaryotes, in particular since *E. coli* and *B. subtilis* are evolutionarily distant. Indeed, the few experimentally known terminators in the Gram-positive *Streptomyces lividans* suggest that there is no need for the stem-loop of a transcriptional terminator to be followed by a T-stretch in this organism [18,19]. Second, we need to make sure that transcriptional terminators can be distinguished from other intercistronic sequences, rather than intracistronic or random sequences, in particular since stem-loop structures in intercistronic sequences may serve other biological functions such as mRNA processing or transcription factor binding. Third, the operon prediction will be reliable only if the fraction of Rho-terminated operons is sufficiently small. We note that previous attempts at terminator prediction in *B. subtilis* [20] and *Synechococcus* sp. WH8102 [21,22] for the purpose of operon prediction were unsuccessful.

Here, we analyzed 463 experimentally known terminating sequences in *B. subtilis* in order to discover their deciding properties. Using a set of 567 experimentally known non-terminating sequences, occurring between genes in the same operon, we derived a decision rule to distinguish between terminating and non-terminating sequences in *B. subtilis*. We show that this decision rule is also valid for other *Firmicutes,* which allowed us to reliably predict their operon structure from the DNA sequence, even in the absence of experimentally known operons in these organisms.

## Results

### Statistical Properties of Rho-Independent Terminators in *B. subtilis*

We created a set of 463 known terminating sequences and 567 known non-terminating sequences in *B. subtilis* by collecting experimentally identified operons from the literature. A stem-loop structure followed by a T-stretch, indicative of a Rho-independent terminator, was found in 425 of the 463 terminating sequences. Here, we analyze these transcriptional terminators and compare their statistical properties with those for the evolutionarily distant *E. coli,* for which 148 proposed and experimentally identified terminators were analyzed previously [16]. The experimentally known operons in *B. subtilis,* the corresponding terminator sequences, and the supporting experimental evidence are available from the DBTBS database [4], as well as in Datasets S1–S3.

**Figure 1:**
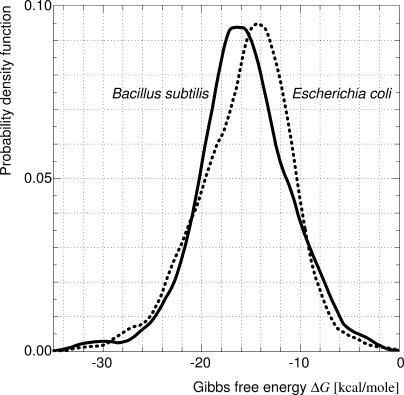
Distribution of the Gibbs Free Energy of Stem-Loop Formation The distribution is calculated from 425 experimentally identified transcriptional terminators in *B. subtilis.* The dotted curve shows the distribution for *E. coli,* as calculated from 147 previously collected Rho-independent terminator sequences in this organism [16].

**Figure 2:**
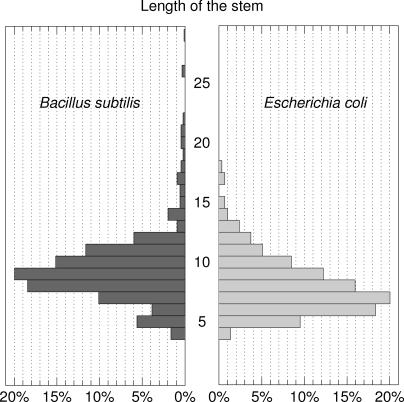
Distribution of the Length of the Stem in Nucleotides The distribution is calculated from 425 transcriptional terminators in *B. subtilis* and 147 previously published Rho-independent terminators in *E. coli* [16].

**Figure 3:**
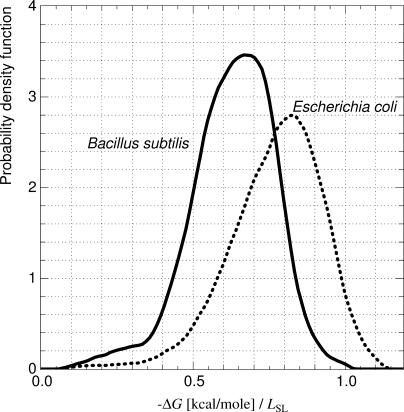
Distribution of Gibbs Free Energy of Stem-Loop Formation, Divided by the Length of the Stem-Loop Structure in Nucleotides The distribution is calculated from 425 transcriptional terminators in *B. subtilis,* and 147 previously published Rho-independent terminators in *E. coli* [16].

Figure 1 shows the distribution of the Gibbs free energy Δ*G* of stem-loop formation at 25 °C. The distribution shows a peak around −16 kcal/mole, compared to −14 kcal/mole for *E. coli* [16]; the extent of the distribution is comparable to *E. coli*. The distribution of the length of the stem, shown in Figure 2, reveals that the transcriptional terminators in *B. subtilis* tend to have relatively long stems: 75% of the stems have a length of 9 ± 2 in *B. subtilis,* whereas in *E. coli* 75% have a length of 7 ± 2. As a result, the density of the Gibbs free energy, calculated by dividing the free energy by the number of nucleotides in the stem-loop structure, is somewhat lower in *B. subtilis* than in *E. coli* (Figure 3). As in *E. coli,* the high density of the Gibbs free energy is made possible by a high G+C content of 62.4% of the stem (78.2% in *E. coli*), compared to an average 36.3% G+C fraction in *B. subtilis* non-coding regions.

**Figure 4:**
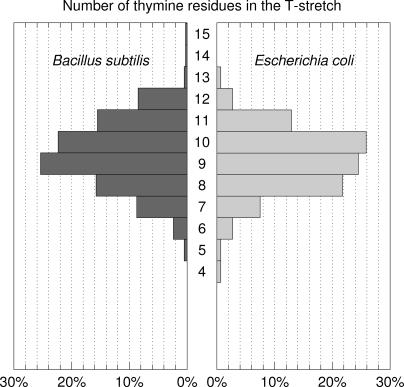
Distribution of the Number of Thymine Residues in the 15 Base Pair T-Stretch following the Stem Loop The distribution is calculated from 425 transcriptional terminators in *B. subtilis,* and 147 previously published Rho-independent terminators in *E. coli* [16].

Figure 4 shows the distribution of the number of thymine residues in a 15−base pair T-stretch following the stem-loop. This distribution, with a median of nine thymine residues, is similar to the distribution previously found for *E. coli* [16], for which the median is equal to ten.

**Figure 5:**
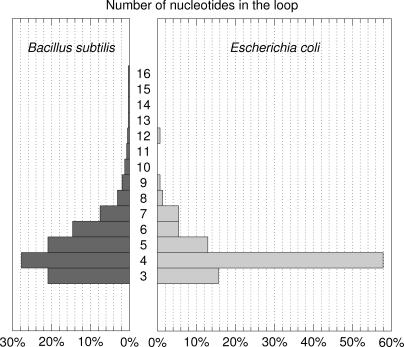
Distribution of the Number of Residues in the Loop of Rho-Independent Terminators The distribution is calculated from 425 transcriptional terminators in *B. subtilis* and 147 previously collected Rho-independent terminator sequences in *E. coli* [16].

To characterize the stem-loops of *B. subtilis* Rho-independent terminators in more detail, we consider the characteristics of the stem and the loop separately. As shown in Figure 5, about 70% of the loops consist of 4 ± 1 nucleotides, compared to 85% for *E. coli*. Whereas tetranucleotides are most abundant (28% of the total), they are not as ubiquitous as in *E. coli,* where they represent 55% of the total.

The tetranucleotides GAAA and TTCG were especially prominent among *E. coli* loops [16]. We did not find this tendency for *B. subtilis,* where TTT, AAT, TGA, and AAAA occurred most often. Also, we did not find evidence that the words GCGGG, GCGGGG, and GGCCC appear most often in the 3′ arm of the stem-loop, as was found for *E. coli* [16]. Instead, we found the words GGCAG (19 times) and GCAGG and TCCGG (17 times each); GCGGG appeared 11 times, GGCCC once, and GCGGGG not at all. The loop is usually closed by a 5′-C-G-3′ pair (35.5% of the loops in *B. subtilis* Rho-independent terminators), although not as often as in *E. coli,* where they constitute 59% of the loop closing pairs.

We note that 82 out of 148 previously analyzed Rho-independent terminators in *E. coli* were proposed in the literature, but were not experimentally verified [16]. Hence, the set of *E. coli* terminators may be biased towards more typical cases.

In many terminators, the T-stretch following the stem-loop structure can base-pair to a complementary sequence in front of the stem-loop, suggesting that the T-stretch may form part of the stem-loop structure. This is particularly evident in stem-loop structures that act as transcriptional terminators on both strands of the DNA. Here, the T-stretch can base-pair to an A-stretch in front of the stem-loop, which acts as a T-stretch for transcription in the opposite direction. In the derivation of the decision rule below, we found that allowing the T-stretch to base-pair to the sequence upstream of the stem-loop did not improve the prediction accuracy. In this paper, we therefore limited the stem-loop structure to the sequence upstream of the T-stretch.

### Position of Transcriptional Terminators in *B. subtilis*

**Figure 6:**
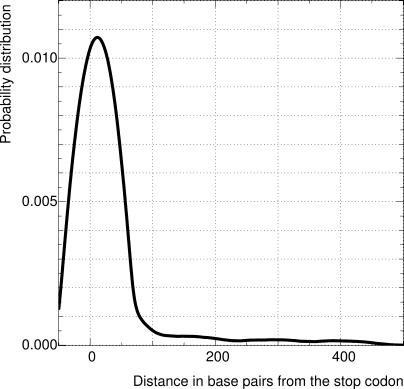
Distribution of the Position of *B. subtilis* Rho-Independent Terminators with Respect to the Stop Codon of the Last Gene in the Operon The distance between the first nucleotide of the stem-loop and the last nucleotide of the stop codon is shown.

As shown in Figure 6, transcriptional terminators are typically located closely downstream of the stop codon of the last gene in the operon and often even partially overlap the gene. Out of 425 experimentally known Rho-independent terminators, 395 are located within 100 base pairs downstream of the stop codon. Of the remaining 30 genes, 14 are immediately followed by a convergently transcribed gene on the opposite strand, such that their 3′ ends are very close to each other or even overlap, leaving little space to fit a Rho-independent transcriptional terminator. As it is difficult to reconcile the requirements of the terminator and the coding region of the downstream gene, the terminator may be located much further downstream in such cases. For example, a Northern blotting experiment [23,24] showed that the terminator of the gene *yxlG* is located 1,136 base pairs downstream of the stop codon, inside the coding region of the convergently transcribed *yxlH* gene, whose 3′ end overlaps for eight base pairs with that of *yxlG*.

In the genome-wide search for transcriptional terminators in *B. subtilis,* described below, we found 466 putative terminators located more than 100 base pairs downstream of the stop codon. Of these, 54 were followed by a convergently transcribed gene with less than 20 base pairs between the stop codons.

A large number of base pairs between the stop codon and the terminator may suggest the presence of a currently unidentified open reading frame. Coding regions highly homologous to known or hypothetical proteins in other organisms were found between the stop codon and the putative terminator of the *B. subtilis* genes *yxiT, fliT, ypbS, metA,* and *ypfD*. These coding regions may correspond to currently unidentified genes in *B. subtilis,* or to genes recently discarded from the *B. subtilis* genome. In the latter case, the position of the terminator may not have stabilized yet.

### Prediction of Transcriptional Terminators in *B. subtilis*

For *E. coli,* the following decision rule was derived previously [16]


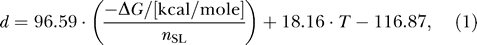


where *T* is the score for the thymine stretch, Δ*G* is the Gibbs free energy of stem-loop formation in kcal/mole, and *n*_SL_ is the number of nucleotides in the entire stem-loop structure. The numerical values for the coefficients were found by fitting this equation to maximize the difference between transcriptional terminators and intracistronic sequences.

The T-stretch score *T* was calculated as follows


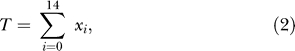


where *x*_0_ = 0.9, and *x_i_* = 0.9 · *x_i_*_−1_ if the *i* ^th^ nucleotide is a thymine, and *x_i_* = 0.6 · *x_i_*_−1_ otherwise [16]. To avoid the usage of ad-hoc parameters, instead we use an exponentially decaying function for the T-stretch


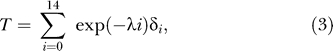


where *δ_i_* is one if the *i* ^th^ nucleotide is a thymine, and zero otherwise. The parameter *λ* is fitted from the experimentally known transcriptional terminators.

Using a logistic regression model to fit this formula to the 463 known terminating sequences and 567 non-terminating sequences in *B. subtilis,* we found the decision rule


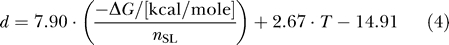


with *λ* = 0.144. This decision rule resulted in a sensitivity of 93.95% and a specificity of 94.36% in predicting transcriptional terminators in *B. subtilis*. The previously proposed T-stretch scoring function (Equation 2) resulted in a slightly lower sensitivity and specificity. As in the case of *E. coli* [16], we found that dividing the Gibbs free energy of stem-loop formation by the length of the stem-loop structure is slightly more accurate than using the Gibbs free energy directly.

As our prediction rule considers only Rho-independent terminators while the training set contains all experimentally known terminating sequences, the high prediction accuracy of about 94% suggests that Rho-dependent terminators account for about 6% or less in *B. subtilis*. Whereas false negatives may also be due to inaccuracies in the numerical values of the parameters in the decision rule (Equation 4), this is unlikely to play a major role. First, the scores *d* of the known terminators follow a bell-shaped distribution (Figure S1) with a tail for negative *d,* such that an imprecision in the numerical values of the decision rule will not strongly affect the accuracy. Second, for most of the false negative predictions we were not able to find any stem-loop structures near the discriminant line *d* = 0 that might conceivably function as a Rho-independent terminator.

A more likely cause of false negative predictions is the presence of Rho-dependent terminators, as well as imperfections in the list of experimentally known operons. For example, some of the false-positive predictions show very clear terminator structures, which may represent read-through terminators or terminator/anti-terminator structures that have not yet been identified experimentally. Partial continuation of transcription at read-though terminators may be regulated or depend on cellular conditions, and cannot always be detected easily in a given experiment. Furthermore, it is sometimes difficult to determine if the 3′ end of an mRNA molecule is produced by transcriptional termination, by mRNA degradation, or by an mRNA processing event, in particular because mRNA processing sites may also be characterized by stem-loops [25,26].

**Figure 7:**
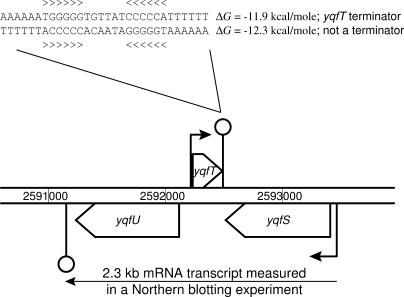
The *yqfSU* Operon in *B. subtilis* Consists of the Two Genes *yqfS* and *yqfU,* Separated by the Intervening Gene *yqfT,* Located on the Opposite Strand The terminator sequence downstream of *yqfT* is virtually identical to the complementary sequence on the opposite strand. However, a Northern blotting experiment [27] revealed that the complementary sequence does not act as a transcriptional terminator. Arrows indicate transcription start sites; stem-loops represent transcriptional terminators.

In some cases, the Gibbs free energy, together with the properties of the T-stretch, does not suffice to predict if a stem-loop terminates transcription. Figure 7 shows the example of the *yqfSU* operon in *B. subtilis*. This operon is unusual, as its two genes are separated by a gene *(yqfT)* that is transcribed in the opposite direction. As *yqfT* is followed by two genes *(yqfS* and *yqfR)* on the opposite strand, we expect *yqfT* to be transcribed monocistronically. Indeed, we find a strong Rho-independent terminator immediately downstream of *yqfT*. Except for the loop sequence, the complementary sequence on the opposite strand is identical to the *yqfT* terminator. However, a Northern blotting experiment [27] revealed that *yqfS* and *yqfU* are transcribed together. Hence, the stem-loop structure terminates transcription on the forward strand, but not on the reverse strand, in spite of the similarity of the stem-loop structure and T-stretch on the two strands. Intervening genes on the opposite strand of DNA were also found for the experimentally known operons *yflMK* (with intervening gene *yflL*), *yfhQ-fabL-sspE* (with intervening gene *yfhS*), and *yqxD-dnaG-sigA* (with *antE* overlaying *dnaG* on the opposite strand).

### Prediction of Transcriptional Terminators in Other Bacteria

As the decision rule (Equation 4) is quite accurate in predicting transcriptional terminators, and hence transcriptional units, in *B. subtilis,* the question arises if the same decision rule can be applied to other organisms related to *B. subtilis*. Very few transcriptional terminators have been identified experimentally in other prokaryotes (except for *E. coli*), making it difficult to verify their conservation in general, or to assess the accuracy of the decision rule when applied to other organisms. However, genes followed by downstream genes on the opposite strand are very likely the last gene in the (mono- or polycistronic) transcription unit, and must therefore be followed by a transcriptional terminator. Hence, we can create a positive set of transcriptional terminators, even in the absence of experimentally known operons, by collecting all genes in the genome that are followed by genes on the opposite strand. As a few examples exist in which a single gene is located between the genes of an operon on the opposite strand, as shown above, we require that a gene is followed by at least two genes on the opposite strand for inclusion in the positive sample set.

The construction of the positive set of transcriptional terminators is based on the assumption that their properties do not depend on whether the downstream gene is on the same strand or the opposite strand of DNA. Creating such a set of positive examples is more difficult for operon prediction based on the intergenic distance [2,3], which is likely to depend on whether two neighboring genes are on the same strand of DNA.

To assess the sensitivity of terminator prediction in other organisms, we apply the decision rule (Equation 4) to the downstream sequence of all genes in the positive set, and count how often it can detect the presence of a transcriptional terminator. The validity of the decision rule can be verified further by analyzing the properties of the predicted transcriptional terminators.

The value for the sensitivity calculated in this manner depends on both the effectiveness of the decision rule in detecting Rho-independent terminators, and the relative importance of Rho-independent terminators with respect to other (possibly Rho-dependent) mechanisms of transcriptional termination. A high sensitivity indicates that Rho-independent termination is dominant in the organism, and that the decision rule effectively finds the Rho-independent terminators. A low sensitivity can arise if the organism has a large number of transcriptional terminators that are not Rho-independent, or if the decision rule is not a valid description of the Rho-independent terminators in that organism.

**Figure 8:**
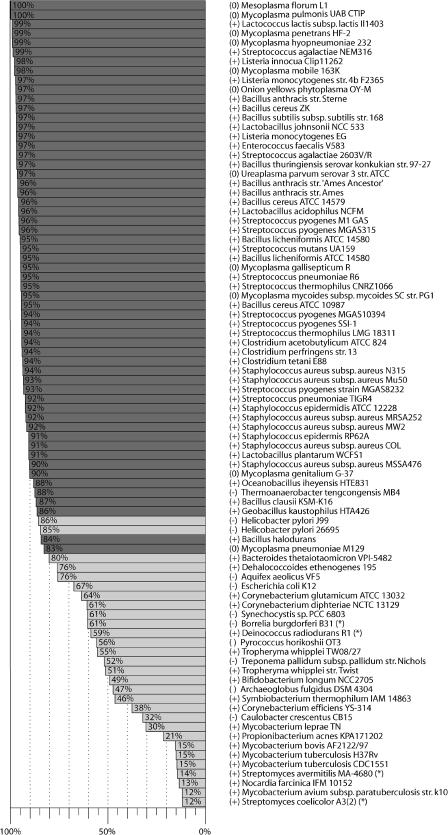
Sensitivity of Predicting Transcriptional Terminators, Evaluated for 57 *Firmicutes* and 29 Other Bacterial Species *Firmicutes* are shown in dark gray; other bacterial species are shown in light gray. (+), (−), or (0) in front of the organism name denotes that the organism is Gram-positive, Gram-negative, or lacks a cell wall, respectively.

The sensitivity of the decision rule (Equation 4) was assessed in the complete genomes of 57 Gram-positive and Gram-negative organisms belonging to the *Firmicutes* phylum, and 19 Gram-positive and 10 Gram-negative bacteria outside of the *Firmicutes* phylum. Figure 8 shows that the prediction rule finds more than 90% of the transcriptional terminators for most *Firmicutes;* on average, the sensitivity is 94.4%. A lower prediction sensitivity, between 80% and 90%, was found for the *Bacillaceae Bacillus halodurans, B. clausii, Oceanobacillus iheyensis, Thermoanaerobacter tengcongensis,* and *Geobacillus kaustophilus*. For organisms outside of the *Firmicutes* phylum, the prediction accuracy is considerably lower.

For *B. subtilis,* the decision rule found a Rho-independent terminator downstream of 97.2% of genes followed by at least two genes on the opposite strand. This indicates an even lower bound on the fraction of genes in *B. subtilis* whose transcription is terminated by Rho, and suggests that perhaps half of the 6% false positives found above are due to inaccuracies in the set of collected terminating and non-terminating sequences.

Tables S1–S3 show the statistical properties of the predicted terminator sequences in more detail. The transcriptional terminators of *Bacillus clausii* and *Geobacillus kaustophilus* are characterized by a slightly lower number of thymine residues in the T-stretch. The other three *Firmicutes* with lower prediction accuracies have a slightly lower Gibbs free energy density Δ*G/L*_SL_ of around −0.54 kcal/mole/nucleotide due to a longer average stem length, particularly for *Oceanobacillus iheyensis* with an average stem length of 11.5 nucleotides. In comparison, the density of the Gibbs free energy in *B. subtilis* is −0.644 kcal/mole, with an average stem length of 9.1 nucleotides.

The transcriptional terminators of the *Staphylococci, Clostridia, Lactobacilli, Lactococci,* and *Streptococci* also have slightly lower values for the Gibbs free energy density compared to *B. subtilis*. For *Lactococcus lactis* subsp. *lactis,* this is compensated for by a larger number of thymine residues in the T-stretch. *Enterococcus faecalis* has an average Gibbs free energy density almost equal to that of *B. subtilis,* in spite of a relatively long stem of 11.9 nucleotides on average.

Modest prediction sensitivities of 90.20% and 82.89% were found for *Mycoplasma genitalium* and *Mycoplasma pneumoniae,* respectively, while higher sensitivities were obtained for other *Mollicutes,* with *Mesoplasma florum L1* and *Mycoplasma pulmonis UAB CTIP* reaching 100%. The transcriptional terminators are characterized by a low Gibbs free energy density of stem-loop formation, ranging from −0.276 kcal/mole/nucleotide for *Mycoplasma hyopneumoniae* to −0.497 kcal/mole/nucleotide for *Mycoplasma gallisepticum,* which explains the previous result (based on the RNA folding energy) that Rho-independent termination plays an insignificant role in these organisms [14]. However, as shown in Table S3, a larger number of thymine residues in the T-stretch ensures that the sensitivity of terminator prediction is high in spite of the lower Gibbs free energy density in *Mollicutes*. In contrast to previous work [14], our results therefore suggest that Rho-independent termination represents the main mode of transcriptional termination in *Mollicutes*. We must mention, however, that based on the predicted transcriptional terminators we found an unusually high number of genes per operon of 3.8 for *Mycoplasma genitalium,* compared to around two for other *Firmicutes,* suggesting that some terminators were missed in the prediction. However, since no Rho protein has been identified in *Mycoplasma genitalium* [8], these cannot be Rho-dependent terminators, unless perhaps Rho proteins are imported from the host organism (in which *Mycoplasmas* live) into the bacterial cell.

The high prediction sensitivities found in the *Firmicutes* suggest that the mechanism of transcriptional termination is conserved in this phylum, both in terms of the validity of the decision rule (Equation 4) to detect Rho-independent terminators, as well as the predominance of Rho-independent transcriptional termination compared to Rho-dependent termination. This allows us to perform an accurate genome-wide prediction of transcriptional terminators, and hence transcriptional units, of the 57 fully sequenced bacterial species of the *Firmicutes* phylum. The full set of transcriptional terminators predicted in a genome-wide search in these 57 *Firmicutes* is available in Dataset S4.

As the separation between Gram-positive and Gram-negative bacteria is one of the most basic divisions in the phylogeny of prokaryotes, one may expect that the decision rule (Equation 4) applies to all Gram-positive organisms. However, as shown in Figure 8, the decision rule detects considerably fewer terminators for Gram-positive bacteria outside of the *Firmicutes* phylum, with sensitivities around 50% for most organisms and as low as 11.8% for *Mycobacterium avium* subsp. *paratuberculosis*. As shown in Table S2, the statistical properties of the predicted terminators tend to deviate more than within the *Firmicutes* group, with longer stems and fewer thymine residues in the T-stretch. The decision rule may therefore be inappropriate for organisms other than *Firmicutes*.

For comparison, Figure 8 also shows the prediction sensitivity for some well-studied Gram-negative organisms. Except for *Helicobacter pylori,* with a sensitivity of about 85%, these sensitivities are generally lower than in *Firmicutes,* ranging from 32% in *Caulobacter crescentus* to 76% for *Aquifex aeolicus*. For *E. coli,* a sensitivity of 67% was found, suggesting a considerable role for Rho-dependent transcriptional termination in this organism.

**Figure 9:**
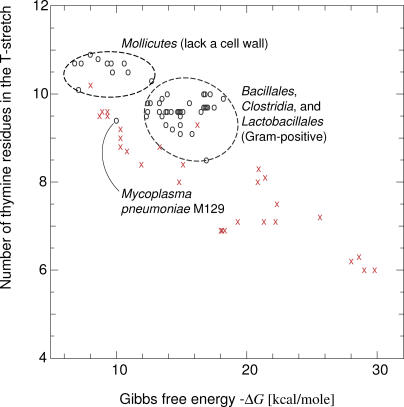
Average Gibbs Free Energy of Stem-Loop Formation and the Average Number of Thymine Residues in the T-Stretch These are calculated from the predicted Rho-independent terminators in the 82 bacterial species we consider. Circles represent organisms belonging to the *Firmicutes* phylum; crosses represent other bacterial species.

Perhaps surprisingly, these prediction accuracies suggest that the mechanism of transcriptional termination is conserved in the *Firmicutes* phylum, but not for Gram-positive bacteria in general. To study the conservation of the properties of Rho-independent transcriptional terminators, we calculated the average Gibbs free energy and the average number of thymine residues in the T-stretch from the predicted terminators in these organisms. Figure 9 shows the position for each organism in this two-dimensional space. The *Firmicutes* appear in two conserved groups, one consisting of the class of *Mollicutes* (including the *Mycoplasmas*) and one consisting of the other classes of the *Firmicutes* phylum, with on average a higher Gibbs free energy of stem-loop formation. As shown in Figure 9, the transcriptional terminators of the bacterial species other than *Firmicutes* display a much larger variation in the properties of their stem-loops and T-stretches. Note, however, that these species may contain a large number of transcriptional terminators that are not Rho-dependent, which affects the accuracy with which the properties of the Rho-independent terminators can be calculated.

## Discussion

As the properties of Rho-independent terminators and the predominance of Rho-independent termination are well conserved within the *Firmicutes* phylum, computationally locating transcriptional terminators from the DNA sequence can be an accurate method to predict the operon structure in these organisms. This is particularly useful for organisms with few experimentally known operons, for which more traditional learning methods cannot be used. We predicted the transcriptional terminators, and hence the operon structure, for 57 prokaryotes belonging to the *Firmicutes* phylum, with an expected sensitivity and specificity of at least 94%.

The comparison of the terminator structures predicted in *Firmicutes* shows that they are conserved as two nearby groups, one corresponding to the *Mollicutes* class (lacking a cell wall), and one corresponding to the other (Gram-positive) classes of *Firmicutes*. From our terminator prediction results in prokaryotes other than *Firmicutes,* we find that the properties of Rho-independent transcriptional terminators are not conserved in bacteria in general, or even in Gram-positive bacteria. In addition, the low prediction sensitivities suggest that other (possibly Rho-dependent) modes of transcriptional termination also play an important role in prokaryotes outside of the *Firmicutes* phylum. However, if Rho is not essential in Gram-positive bacteria in general, as suggested previously [9], then a currently unknown mechanism of transcriptional termination must be present in Gram-positive bacteria other than *Firmicutes*. Also for the cyanobacterium *Synechocystis* sp. strain PCC6803, which apparently lacks the Rho protein altogether [7], we expect the existence of alternative termination mechanisms, as the prediction of Rho-independent transcriptional terminators showed a sensitivity of only 61%.

While Rho-independent termination is dominant in *B. subtilis,* we were not able to find a clear transcriptional terminator for 28 experimentally known operons as well as 15 other genes followed by two or more genes on the opposite strand. These operons may therefore be followed by a Rho-dependent terminator. The list of experimentally known operons in *B. subtilis* together with the set of predicted transcriptional terminators, both of which are available in the Supporting Information, may therefore contribute to further research of Rho-dependent and other mechanisms of transcriptional termination.

## Materials and Methods

Calculation of the RNA secondary structure was done with Mfold [28,29]. To calculate the Gibbs free energy of formation for the terminator stem-loop structure, we first searched the DNA sequence downstream of a gene to find candidate thymine stretches with a length of 15 base pairs. We required the T-stretch to start with at least two thymine residues. Relaxing this condition did not improve the prediction accuracy, but it significantly increased the running time, which can be more than a day for a complete genome. T-stretches with less than three thymine residues, as well as T-stretches with a *T*-score of less than 2.5, were ignored, as such weak T-stretches are unlikely to function as a terminator even if preceded by a very strong stem-loop. We then let Mfold calculate the RNA secondary structure of the upstream 75 base pairs at a temperature of 25 °C. Usually, Mfold finds more than one stem-loop or other secondary structures in this sequence. If so, we analyzed the output of Mfold to find the first paired nucleotide, and removed it together with the nucleotides further upstream. We then recalculated the secondary structures in this shortened RNA stretch, and repeated until a single stem-loop structure was found. We allowed up to three consecutive nucleotides in the stem-loop to be mismatched or unmatched, as well as a gap of at most three nucleotides between the last base-paired nucleotide in the stem-loop and the start of the T-stretch.

As soon as the sequence upstream of the T-stretch folded as a single stem-loop structure, we found its Gibbs free energy −Δ*G* and applied the decision rule (Equation 4) to determine if it qualified as a Rho-independent transcriptional terminator. If so, the terminator structure and the corresponding Gibbs free energy were saved. If not, we continued with the next candidate T-stretch.

The sequence to be searched for the existence of a transcriptional terminator depends on the position of neighboring genes. We started the search at 25 base pairs upstream of the stop codon, as the stem-loop often partially overlaps the coding region of the gene. The search continued until 500 base pairs downstream of the stop codon. However, if the gene was followed by another gene on the same DNA strand, the search sequence ended at its start codon. The search sequence we chose was considerably larger than in previous studies, as we found that transcriptional terminators located far downstream of the stop codon are not uncommon, while the false-positive rate of terminator prediction is quite low.

To find the decision rule (Equation 4), we searched the downstream sequence of 463 known terminating sequences and 567 known non-terminating sequences, retaining all terminator-like structures for each sequence and the corresponding Gibbs free energy Δ*G*. We used the Newton-Raphson method to iteratively update the decision rule, for each sequence choosing the highest-scoring terminator-like structure among the candidates during the iteration, until no further improvement could be achieved. The iteration started from a random initial score for the numerical parameters in Equation 4; the final values for the parameters were found to be independent of the initial random choice. The parameter *λ,* representing the fall-off in the scoring function for the T-stretch, was determined from the positive examples only.

Finding the appropriate sections of the DNA sequence, running the Mfold program, analyzing its results, and running the Newton-Raphson iteration was automated using the Python scripting language [30]. The Python script is available upon request from the author.

The analysis of transcriptional terminators in *E. coli* is based on a previously collected set of 148 Rho-independent terminators in that organism [16]. As one of the terminator sequences appears to be missing from the published list, the analysis presented here is based on 147 terminator sequences only.

## Supporting Information

Dataset S1 List of Experimentally Identified Operons in *B. subtilis*, Annotated Manually Each row describes one operon, with the following columns:
The genes the operon consists of
The operon name
The position of the Rho-independent transcriptional terminator, if present
The Gibbs free energy of stem-loop formation, in kcal/mole, evaluated at 25 °C using Mfold
The RNA sequence of the terminator structure, with <left>, </left> and <right>, </right> delimiting the left and right arm of the stem-loop
The experimental evidence for the operon
A list of literature references with PubMed numbers
Optionally, some additional comments on the operon structure or the experimental evidence
This information is also available through the DBTBS database of transcriptional regulation in *B. subtilis* (http://dbtbs.hgc.jp).
(254 KB TXT)

Dataset S2 List of Genes and Experimentally Known Operons in *B. subtilis,* in a Machine-Readable Form (220 KB TXT)

Dataset S3 List of 425 Rho-Independent Terminators, Deduced from the List of Experimentally Known Operons in *B. subtilis*
Each row in this list contains the following columns:
The operon name
Last gene in the operon
Position of the first nucleotide in the stem-loop structure with respect to the stop codon of the last gene in the operon
The Gibbs free energy of stem-loop formation, in kcal/mole, evaluated at 25 °C using Mfold
The RNA sequence of the terminator structure, with <left>, </left> and <right>, </right> delimiting the left and right arm of the stem-loop
(39 KB TXT)

Dataset S4 The Predicted Transcriptional Terminators in 57 Bacterial Organisms of the *Firmicutes* Phylum (2.4 MB TGZ)

Table S1 Terminator Prediction in Bacterial Species in the *Firmicutes* Phylum (5 KB TXT)

Table S2 Terminator Prediction in Gram-Positive Bacteria other than *Firmicutes*
(2 KB TXT)

Table S3 Terminator Prediction in Gram-Negative Bacteria outside the *Firmicutes* Phylum (1 KB TXT)

Figure S1 Distribution of the Discriminant Scores *d* as Calculated from the Decision Rule for Known Terminators in *B. subtilis*
(197 KB EPS)
